# Prostate-Specific Membrane Antigen-Targeted Site-Directed Antibody-Conjugated Apoferritin Nanovehicle Favorably Influences *In Vivo* Side Effects of Doxorubicin

**DOI:** 10.1038/s41598-018-26772-z

**Published:** 2018-06-11

**Authors:** Simona Dostalova, Hana Polanska, Marketa Svobodova, Jan Balvan, Olga Krystofova, Yazan Haddad, Sona Krizkova, Michal Masarik, Tomas Eckschlager, Marie Stiborova, Zbynek Heger, Vojtech Adam

**Affiliations:** 1 Department of Chemistry and Biochemistry, Mendel University in Brno, Zemedelska 1, Brno, CZ-613 00 Czech Republic; 2 Central European Institute of Technology, Brno University of Technology, Purkynova 123, Brno, CZ-612 00 Czech Republic; 3 Department of Pathological Physiology and Department of Physiology, Faculty of Medicine, Masaryk University, Kamenice 753/5, Brno, CZ-625 00 Czech Republic; 4 TESCAN ORSAY HOLDING a.s., Libusina trida 863/21, Brno, CZ-623 00 Czech Republic; 5 Department of Pediatric Hematology and Oncology, 2nd Faculty of Medicine, Charles University and University Hospital Motol, V Uvalu 84/1, Prague 5, CZ-150 06 Czech Republic; 6 Department of Biochemistry, Faculty of Science, Charles University, Hlavova 2030/8, Prague 2, CZ-128 43 Czech Republic

## Abstract

Herein, we describe the *in vivo* effects of doxorubicin (DOX) encapsulated in ubiquitous protein apoferritin (APO) and its efficiency and safety in anti-tumor treatment. APODOX is both passively (through Enhanced Permeability and Retention effect) and actively targeted to tumors through prostate-specific membrane antigen (PSMA) *via* mouse antibodies conjugated to the surface of horse spleen APO. To achieve site-directed conjugation of the antibodies, a HWRGWVC heptapeptide linker was used. The prostate cancer-targeted and non-targeted nanocarriers were tested using subcutaneously implanted LNCaP cells in athymic mice models, and compared to free DOX. Prostate cancer-targeted APODOX retained the high potency of DOX in attenuation of tumors (with 55% decrease in tumor volume after 3 weeks of treatment). DOX and non-targeted APODOX treatment caused damage to liver, kidney and heart tissues. In contrast, no elevation in liver or kidney enzymes and negligible changes were revealed by histological assessment in prostate cancer-targeted APODOX-treated mice. Overall, we show that the APO nanocarrier provides an easy encapsulation protocol, reliable targeting, high therapeutic efficiency and very low off-target toxicity, and is thus a promising delivery system for translation into clinical use.

## Introduction

Various potent chemotherapeutic drugs have been developing over the decades. Despite their profound therapeutic efficacy^1^, they cause numerous dose-limiting side effects^2^. Doxorubicin (DOX) is but one example of this phenomenon, where DOX administration leads to arrhythmia or cardiomyopathy caused by the formation of reactive oxygen species and cytochrome *c* release from mitochondria^3^ in up to 26% of patients^4^. To decrease these effects, DOX is often co-administrated with the cardioprotective agent dexrazoxane. However, its cardioprotective abilities are contentious and many patients treated with dexrazoxane have developed secondary malignancies^5^.

To eliminate the challenges of conventional cancer chemotherapy, preferential delivery of anti-cancer drugs to tumor cells is being investigated. This can be achieved using nano-scaled drug-containing particles, which are called nanocarriers^6^. The ideal nanocarrier needs to not only be non-toxic but also biocompatible and biodegradable^7^. These properties are important for both the subjects involved in the treatment and the general public since the nanoparticles are often excreted into waste water and can pose a threat to the environment^8^. Many different materials have been studied for the preparation of drug nanocarriers, both organic and inorganic^9^. Inorganic exogenous materials are usually not biodegradable and can be accumulated in an organism following repeated administration or prematurely captured in organs of the reticuloendothelial system^10^. They can also cause inflammatory response or neurotoxic reactions^8^. Organic exogenous particles also have some drawbacks. Currently, there are two commercially available nanopharmaceuticals containing DOX: Myocet^©^ (DOX in bare liposomes) and Doxil^©^ (DOX in polyethylenglycolated (PEGylated) Stealth^®^ liposomes)^11^. Bare liposomes were found to be recognized by patient’s cytotoxic T cells and removed from the body prior to reaching the tumor site^12^. Although PEGylated liposomes are able to evade the immune cells, their cellular uptake is hampered due to the PEGylation. Moreover, they have been proven to cause palmar-plantar erythrodysesthesia^13^ and pulmonary fibrosis^14^.

In light of these facts, endogenous particles seem more promising; especially those involved in the cellular uptake pathways. They are naturally biocompatible and biodegradable and they also provide easy passage through the cell membranes^15^. These much-needed properties can be provided by ferritins or better apoferritins (APO, demineralized ferritins), ubiquitous proteins with high interspecies sequence homology responsible for the storage and transfer of iron ions^16^. Our previous study^17^, as well as studies of others^18–21^, have proven that site-directed APO could enhance the *in vitro* selectivity of encapsulated cytotoxic drug for cancer tissue, while retaining its potency.

In the present study, we evaluated the prostate cancer-targeted horse spleen APO-encapsulated DOX for the first time in terms of its mechanisms of internalization into tumor cells, prostate tumor attenuation in murine ectopic xenografts and its effects on the off-target organs of the administered mice. The site-directed orientation of targeting antibodies was achieved through protein A-derived heptapeptide, which was attached to 1.3 nm gold nanoparticle-modified APO surface *via* cysteine on its *C*-terminus. Overall, we show that while the nanocarrier was able to attenuate the tumors with slightly lower potency than free DOX, it significantly spared the off-target organs from the unwanted toxic action of free DOX.

## Results

### Elucidating the cellular uptake efficiency and mechanism of APODOX-anti-PSMA

**Figure 1:**
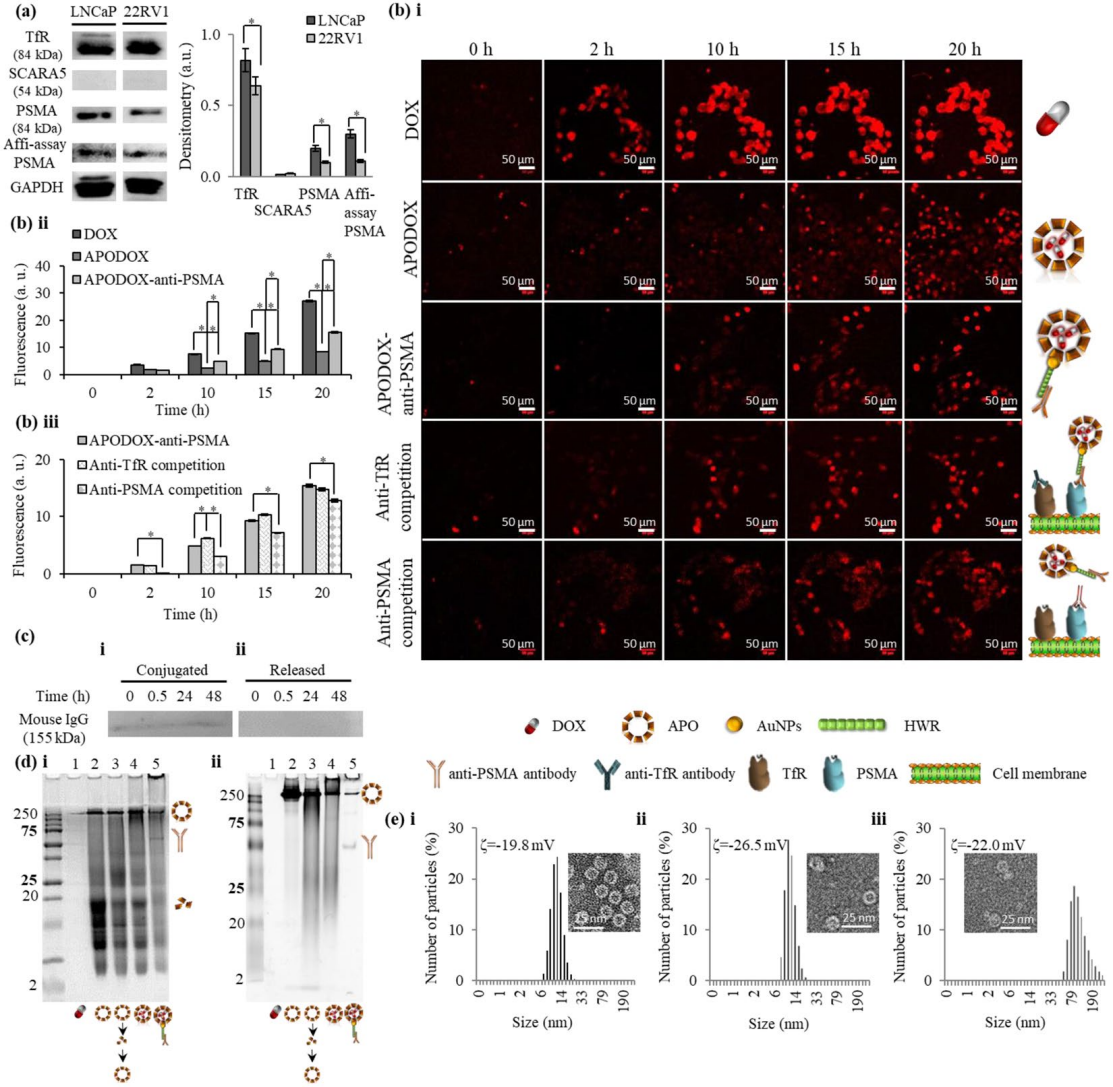
*In vitro* cellular uptake of APODOX-anti-PSMA in prostate cancer cell lines. (**a**) Expression profiles of GAPDH, TfR, SCARA5 and PSMA and affinity binding of APODOX-anti-PSMA to PSMA on LNCaP and 22RV1 prostate cancer cell lines. The individual blots were cropped from different parts of the same membrane (as indicated by dividing white spaces). Right part shows the densitometric analysis of expression of TfR, SCARA5 and PSMA and binding to PSMA relatively compared to the expression of the house-keeping protein GAPDH. The densitometric analyses were performed on uncropped images using AzureSpot software. The values are expressed as means ± standard deviations of independent triplicates. Vertical bars indicate standard deviation. *Indicate significant differences (*p* < 0.05) between the two cell lines. (**b**) DOX fluorescence obtained from continuous quantitative phase imaging of LNCaP cells treated with DOX/APODOX/APODOX-anti-PSMA and the competitive assay between APODOX-anti-PSMA and anti-TfR/anti-PSMA antibodies. (i) DOX fluorescence micrographs obtained at various time points of treatment. (ii) DOX fluorescence values from cells treated with DOX/APODOX/APODOX-anti-PSMA at various time points expressed as means ± standard deviations of six independent measurements. Vertical bars indicate standard deviation. *Indicate significant differences (*p* < 0.05) among the tested groups. (iii) DOX fluorescence values from competitive assay between APODOX-anti-PSMA and anti-TfR/anti-PSMA antibodies expressed as means ± standard deviations of six independent measurements. Vertical bars indicate standard deviation. *Indicate significant differences (*p* < 0.05) between cells pre-treated with antibodies and APODOX-anti-PSMA cellular uptake without competition. (**c**) Stability of mouse anti-PSMA binding to APODOX evaluated by incubation of APODOX-anti-PSMA with 10 mg·mL^−1^ human IgG for 0, 0.5, 24 and 48 h with subsequent detection of mouse antibodies conjugated on APODOX (i) and released into solution (ii). (**d**) 12.5% SDS-PAGE (i) and native-PAGE (ii) gels showing protein content of DOX (1) APO (2), APO after disassembly and reassembly (3), APODOX (4) and APODOX-anti-PSMA (5). Marked are the > 250 kDa APO, ~60 kDa antibody, and ~19 kDa light subunit. (**e**) Size, ζ-potential and TEM micrographs (inset) of APO (i), APODOX (ii) and APODOX-anti-PSMA (iii).

To choose suitable cells for mice xenograft studies, two prostate cancer cell lines, LNCaP and 22RV1, were evaluated based on their ability to bind and internalize APODOX-anti-PSMA (prostate-specific membrane antigen). As cellular uptake of APO through either transferrin receptor 1 (TfR)^22^, T cell immunoglobulin and mucin domain-containing protein-2 (TIM-2)^23^ or scavenger receptor class A member 5 (SCARA5)^22^ is based on this heavy and light chain subunit ratio, their expression was tested. Figure 1a shows the expression profiles of TfR, SCARA5 and PSMA as the most probable preferential targets for APODOX-anti-PSMA binding, as well as the results from Affi-assay, evaluating the binding of APODOX-anti-PSMA to 84 kDa PSMA protein. Densitometric analysis revealed significant differences (*p* < 0.05) between the two tested cell lines, with LNCaP having higher expression of both TfR (1.3× higher) and PSMA (2.0× higher), as well as higher binding of APODOX-anti-PSMA (2.8× higher). Noteworthy, only negligible expression of preferential receptor for L-type ferritins SCARA5 was found on these cell lines.

Hence, we focused our attention on internalization of APODOX-anti-PSMA in LNCaP cells. To determine uptake kinetics, we obtained mean intracellular DOX fluorescence values from continuous quantitative phase imaging (24 h) of treated cells (Fig. 1bi and bii). The significantly (*p* < 0.05) highest uptake was observed for free DOX (3× higher fluorescence than for APODOX and 1.7× for APODOX-anti-PSMA after 20 h of treatment). First signs of the cell shrinkage were observed after 1 h treatment with DOX, with membrane budding clearly visible after 4 h of treatment (Supplement 1). APODOX exhibited lower cellular uptake compared to free DOX; however, site-directed surface modification with antibodies favorably influenced the uptake (1.8× higher intracellular fluorescence of APODOX-anti-PSMA than of APODOX, Fig. 1bi and bii). Moreover, the first shrinking cells were visible after 10 h of treatment and cell budding was observed after more than 20 h of treatment with APODOX (Supplement 2). Whereas, the cells treated with APODOX-anti-PSMA also showed first signs of the cell shrinkage after 10 h of treatment but the budding was visible after 14 h, and significantly faster than in the case of APODOX (Supplement 3).

To elucidate the mechanisms of cellular uptake, a competitive assay between APODOX-anti-PSMA and anti-TfR/anti-PSMA antibodies was performed (Fig. 1bi and biii). The competition between SCARA5 and APODOX-anti-PSMA was not studied, due to the very low SCARA5 expression. The competition with anti-TfR antibodies did not inhibit the uptake of APODOX-anti-PSMA and the signs of early and late apoptosis were observed even earlier (cell shrinkage at 5 h of treatment and budding at 12 h– see Supplement 4). However, competition with anti-PSMA antibodies showed significantly lower cellular uptake of APODOX-anti-PSMA (1.2× lower uptake after 12 h of treatment). The cell shrinkage was also observed after 20 h of treatment with budding after 23 h of treatment (Supplement 5). These results suggest that APODOX-anti-PSMA does internalize into cells predominantly through the PSMA.

To obtain insight into the stability of targeting antibody (mouse anti-PSMA antibody) binding to APODOX surface in the blood stream, APODOX-anti-PSMA was incubated with human IgG at plasma concentration for up to 48 h and mouse antibodies were detected on APODOX surface and in the solution at various time points (Fig. 1c). The results showed that the binding was stable for up to 48 h, where the competition with human IgG antibodies did not cause any removal of targeting mouse antibodies from the surface of APODOX.

To evaluate the protein profile of APO, APO after pH-mediated disassembly and reassembly, APODOX and APODOX-anti-PSMA, we resolved the samples on SDS and native PAGE (Fig. 1d). The SDS-PAGE showed the content of ~15 and ~12 kDa proteins in the sample of APO as received from the manufacturer, although these were not observed on native PAGE. The results showed that neither the disassembly/reassembly, nor encapsulation of DOX changed the protein profile of APO, whereas, as expected, changes were observed after the modification with PSMA-targeting antibody.

Size measurements (Fig. 1e) revealed the unchanged 12-nm size of APO after DOX encapsulation while the targeting antibody increased the size of the whole nanoconstruct to 91 nm which was presumably due to a partial aggregation. These results correspond to results obtained from TEM micrographs (Fig. 1e insets), showing antibodies bound on APO surface without further changes to the morphology of assembled APO. The ζ-potential in plasma, ranging from −19.8 mV for APO, to −26.5 mV for APODOX and −22.0 mV for APODOX-anti-PSMA, highlights pronounced stability in biological milieu.

### Effect of treatment on tumor attenuation and plasma biochemistry

**Figure 2:**
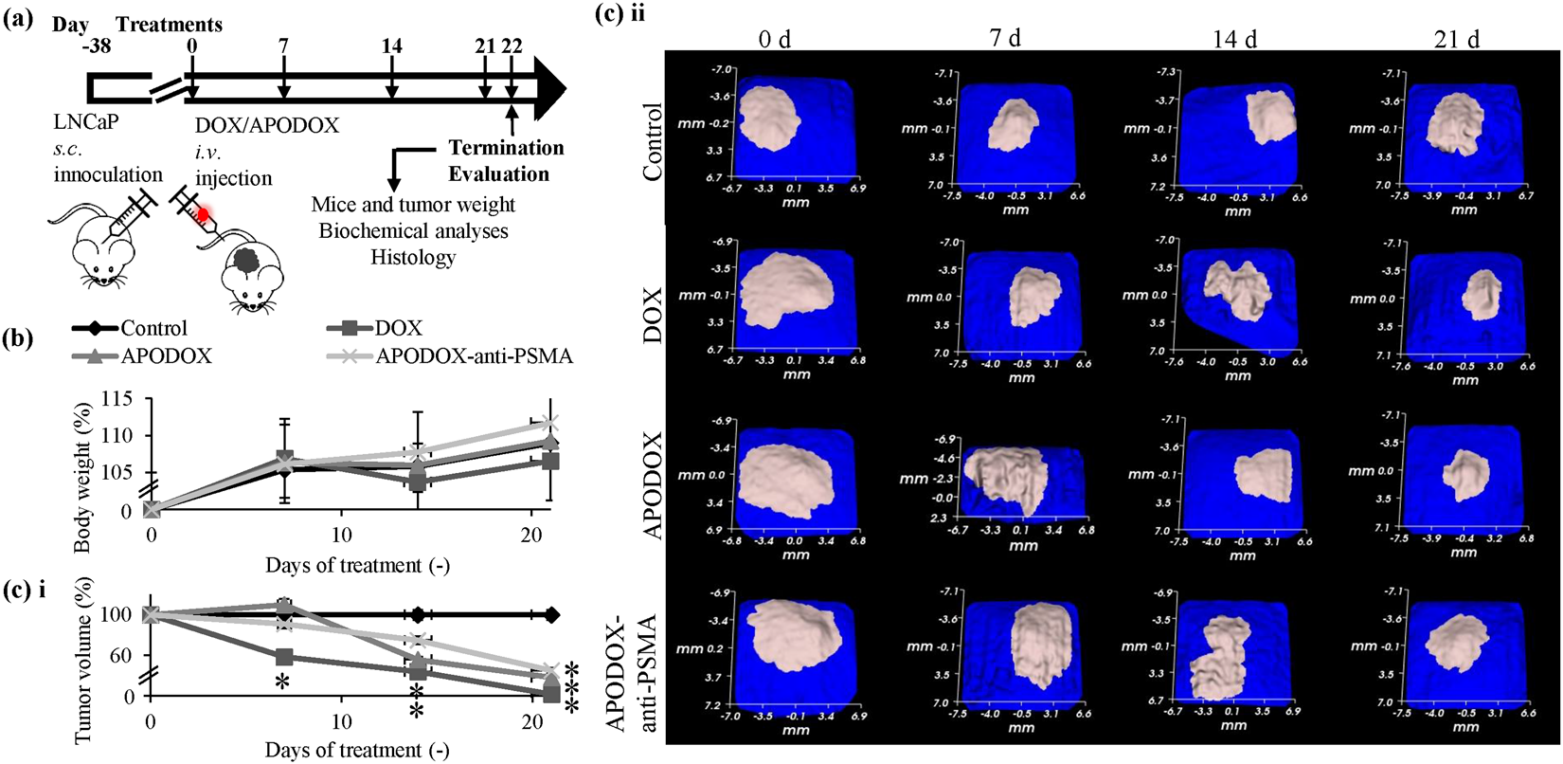
The attenuation of *s.c*. murine LNCaP xenografts treated with DOX/APODOX/APODOX-anti-PSMA. (**a**) Schematic depiction of experimental workflow beginning with the LNCaP cells (5 × 10^6^) *s.c*. inoculation. The schematics of mouse and syringe were adopted under General Public Licence from pixabay.com. (**b**) Average weight of mice determined over the course of experiment with the weight at the start of experiment designated as 100%. (**c**) Changes in tumor size during the experiment. (i) 3D reconstruction of tumors. (ii) Changes in tumor volumes compared to the volume at the start of experiment. The values are expressed as means ± standard deviations of independent triplicates. *Indicate statistically significant differences (*p* < 0.05) when comparing treatments with control (saline).

Next, we proceeded to evaluate the *in vivo* effects of APODOX-anti-PSMA utilizing murine xenografts, focusing on its effects on tumor growth and damage to the off-target organs. *In vitro* cytotoxicity tests on prostate cancer and healthy cell lines were concluded in detail and we refer to results in our previous work^17^. Murine xenografts were induced by *s. c*. inoculation of LNCaP cells. Figure 2a shows the experimental workflow of the *in vivo* experiment, showing the treatment course, termination and subsequent analyses. Although all mice gained weight throughout the course of the experiment, DOX-treated mice showed significant (*p* < 0.05) losses of weight relative to the saline-injected controls (Fig. 2b). No mice died or had to be euthanized prior to the end of the experiment.

The 3D reconstruction of tumors (Fig. 2c) revealed their significant (*p* < 0.05) attenuation in all treated groups. The fastest attenuation was observed in DOX-treated mice with 42 and 78% decrease in tumor mass after 1 and 3 weeks of treatment, respectively. Mice treated for 3 weeks with APODOX and APODOX-anti-PSMA showed decrease in tumor mass by 62 and 55%, respectively.

**Figure 3:**
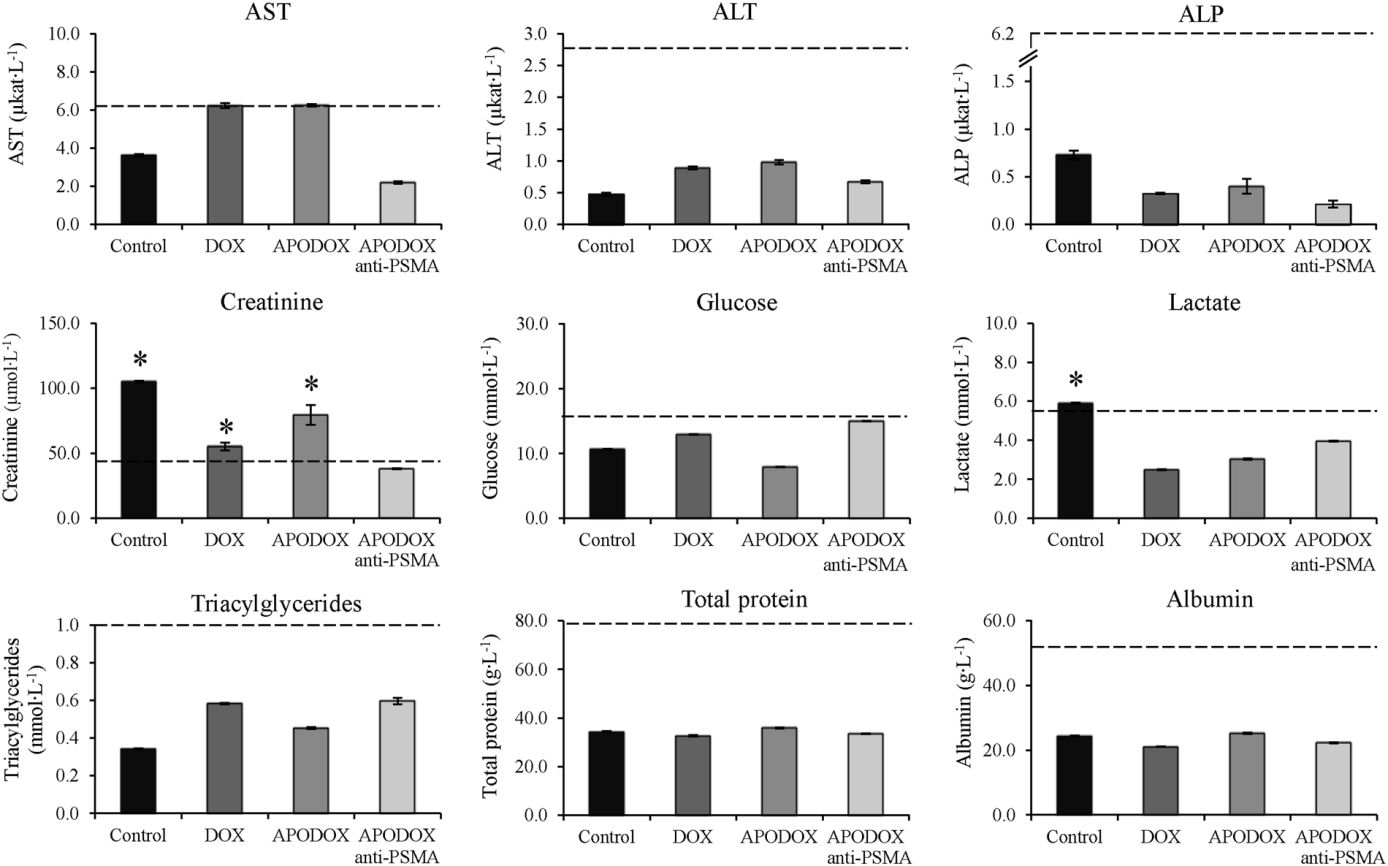
Plasma biochemistry analyses revealing plasma levels of AST, ALT, ALP, creatinine, glucose, lactate, total protein, albumin and triacylglycerides in mice treated with DOX/APODOX/APODOX-anti-PSMA and saline-injected controls. The values are expressed as means ± standard deviations of independent triplicates. Vertical bars indicate standard deviation. Dashed lines indicate upper limit of physiological values for selected plasma markers. *Indicate statistically significant increase (*p* < 0.05) above the physiological limit.

Blood was collected at euthanasia to assess renal and liver function. Various biochemical parameters were tested, including creatinine, alanine aminotransferase (ALT), aspartate aminotransferase (AST), or alkaline phosphatase (ALP) as the results are shown in Fig. 3. The liver function markers ALT and AST were significantly elevated in mice treated with DOX (AST 6.23 µkat∙L^−1^ and ALT 0.89 µkat∙L^−1^) and APODOX (AST 6.25 µkat∙L^−1^, ALT 0.97 µkat∙L^−1^), while mice treated with APODOX-anti-PSMA showed similar values to that of saline-injected mice and within the normal range (AST 2.19 µkat∙L^−1^, ALT 0.67 µkat∙L^−1^ in APODOX-anti-PSMA-treated mice, AST 3.63 µkat∙L^−1^, ALT 0.47 µkat∙L^−1^ in saline-injected mice). No elevation in ALP levels was observed in any of the treated groups. Creatinine levels, as a marker of kidney function, did not show significant elevation in any of the treated groups compared to saline-injected mice. From the data obtained, it follows that no obvious hepatic or renal toxicity was observed in treated mice. Glucose, lactate, proteins or albumin ratio also showed no significant elevation. Triacylglycerides showed slight elevation in all treated groups compared to saline-injected mice.

### Histologic assessment of excised organs

**Figure 4:**
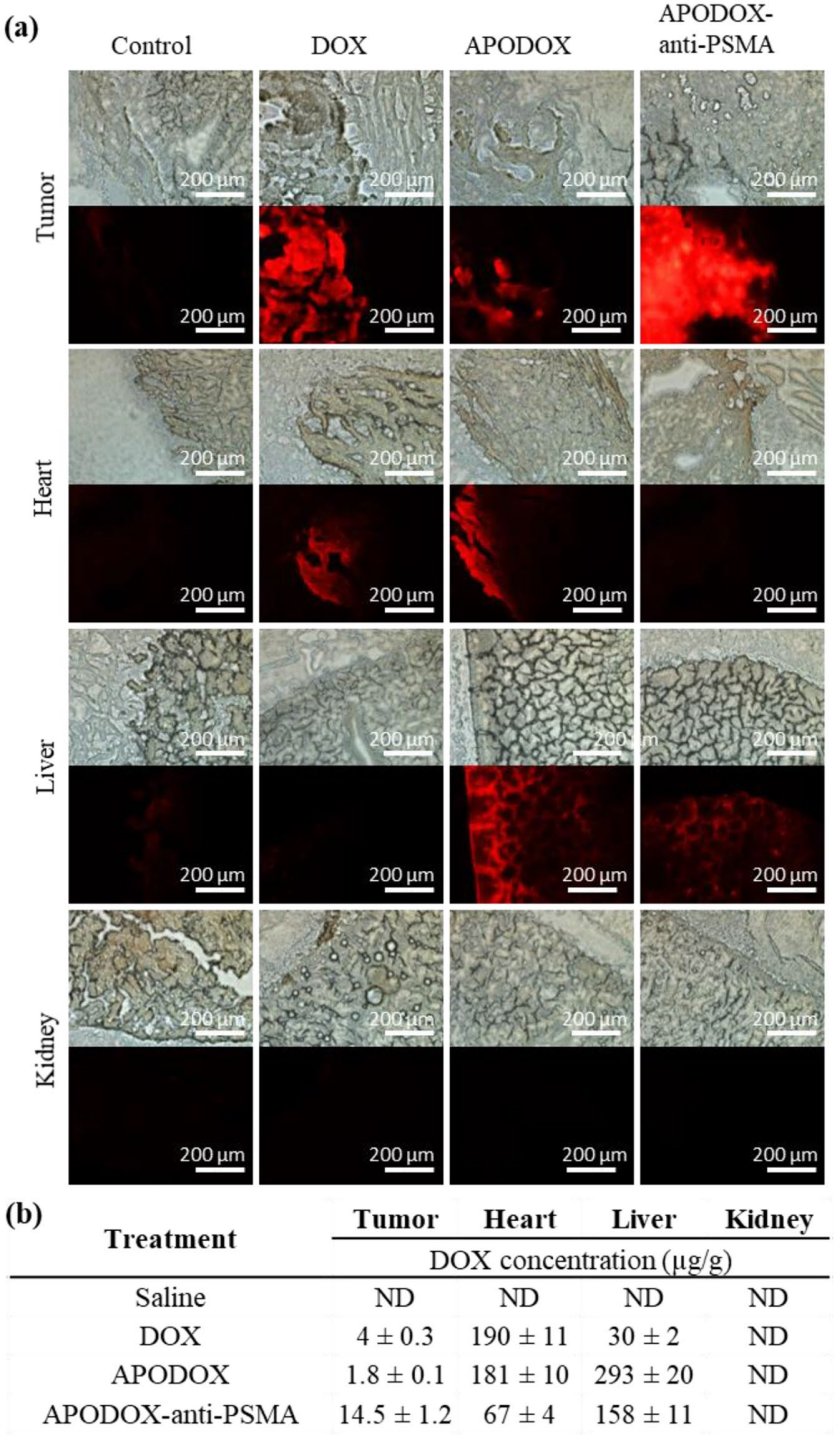
(**a**) Micrographs showing DOX distribution in histological sections of excised tumor, heart, liver and kidney from mice treated with DOX/APODOX/APODOX-anti-PSMA and saline-injected controls. (**b**) DOX distribution in the organ homogenates after extraction by acidified isopropanol.

Heart, liver and kidney were collected from each mouse to test the distribution of the various forms of DOX in off-target organs and their toxicity. To evaluate DOX distribution in the organs, we employed its specific fluorescent properties in the tissue slices (Fig. 4a), as well as in the homogenates after extraction by acidified isopropanol (Fig. 4b). The autofluorescence of all tested organs and their homogenates excised from saline-injected mice was used as a background. DOX-treated mice showed high DOX concentration in tumor and heart, with low concentration in liver and undetectable concentration in kidney. APODOX-treated mice showed low DOX concentration in tumor (2.2× lower than in DOX-treated mice), with still high concentration in heart (1.1× lower than in DOX-treated mice) and connective tissue of liver and undetectable concentration in kidney. Tumors excised from APODOX-anti-PSMA-treated mice showed the highest observed DOX concentration among all the mice groups (3.6× higher than in DOX-treated mice). On the contrary, very low or undetectable concentration of DOX was found either in heart (2.8× lower than in DOX-treated mice) or in kidney of these mice. Low DOX concentration was observed in connective tissue of liver.

**Figure 5:**
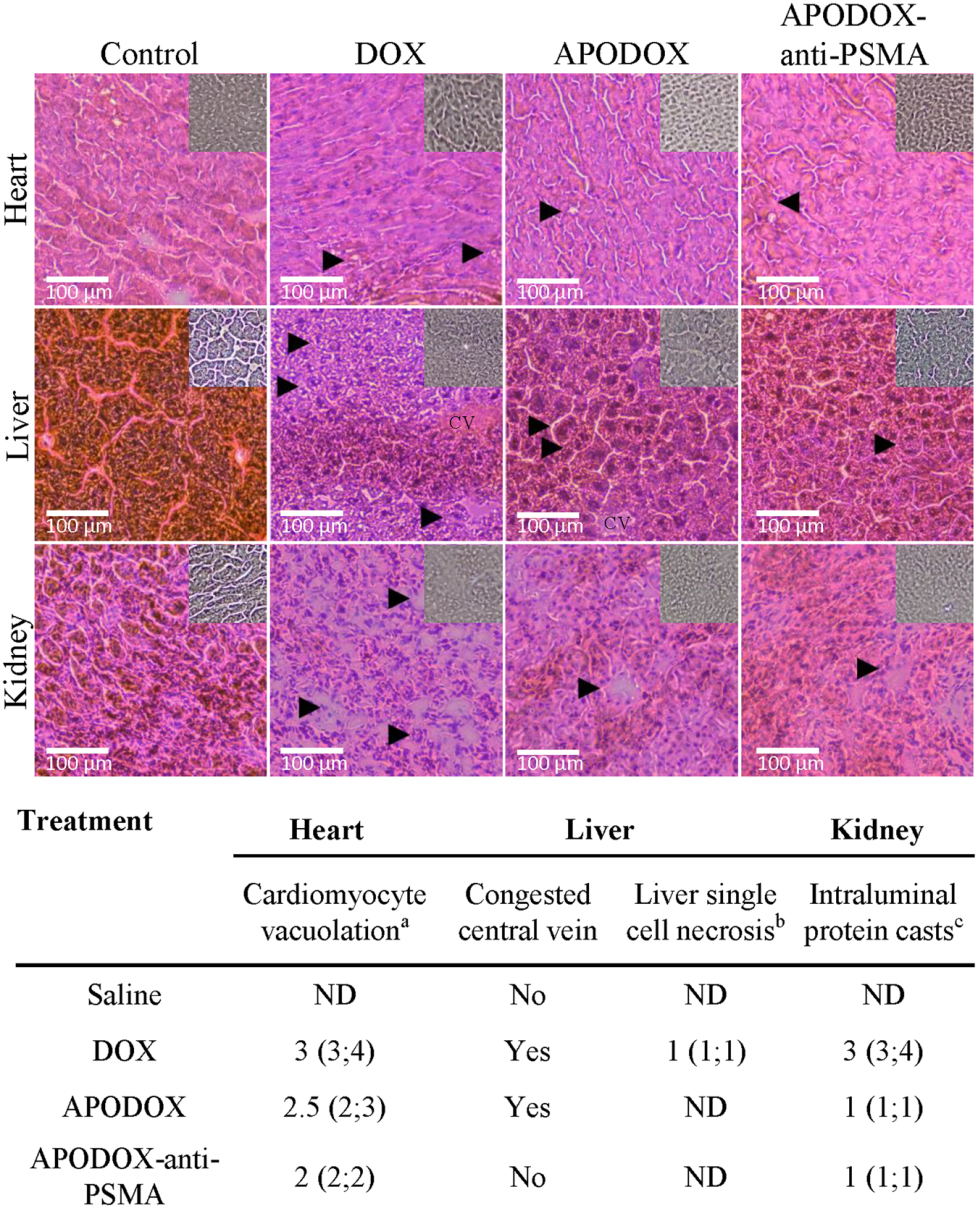
Upper part: H&E-stained tissue section of off-target organs (heart, liver and kidney) collected from mice treated with DOX/APODOX/APODOX-anti-PSMA and saline-injected controls. Black arrows in heart show myocardiocyte vacuolation. Black arrows in liver show centrilobular hepatocyte binucleation. CV shows the congested central vein. Black arrows in kidney show intraluminal protein casts. Insets show distribution of iron detected by Perls’ Prussian blue staining. Lower part: Histopathological findings and severity scores in heart, liver and kidney in mice treated with the various DOX formulations. (**a**) Cardiomyocyte vacuolation (% affected cardiomyocytes): 1 = 1–5%; 2 = 5–10%; 3 = 10–25%; 4 = greater than 25%. (**b**) Liver single cell necrosis: 1 = dead hepatocytes were rarely observed (1 per 10 high power field/400×). (**c**) Intraluminal protein casts: 1 = detected in less than 5% of the tubular profiles; 2 = detected in 5–25% of the tubular profiles; 3 = detected in 25–50% of the tubular profiles; 4 = detected in greater than 50% of the tubular profiles.

The microstructure and iron content were assessed in heart, liver and kidney (Fig. 5 for structure and Fig. 5 inset for iron content). The histological examination of heart sections revealed significant (10–25%) myocardiocyte vacuolation in DOX-treated mice. However, the APODOX and APODOX-anti-PSMA mice showed only slight vacuolization in the heart (5–10% for APODOX and 1–5% for APODOX-anti-PSMA). There was no evidence of changes in heart iron content in any of the tested groups. Histological sections of the liver showed disorganization of the normal appearance, increased liver single cell necrosis and centrilobular hepatocyte binucleation and congested central veins in DOX-treated mice. Increased centrilobular hepatocyte binucleation was also observed in APODOX-treated mice, but only sporadically appeared in APODOX-anti-PSMA treated mice. A slight decrease in liver iron content was also observed in both DOX-and APODOX-treated mice while APODOX-anti-PSMA-treated mice showed similar iron content to saline-injected ones.

Histological assessment of kidney showed the presence of large number of intraluminal protein casts in DOX-treated mice (in 25–50% of tubular profiles), while in mice treated with APODOX and APODOX-anti-PSMA, these protein casts were formed only sporadically (in less than 5% of tubular profiles). Iron was only sporadically found in capillaries of all groups, too. The obtained results show that encapsulation of DOX in prostate cancer-targeted APO lowers the influence of DOX on off-target organs.

### Sequence homology of ferritins from different organisms

**Figure 6:**
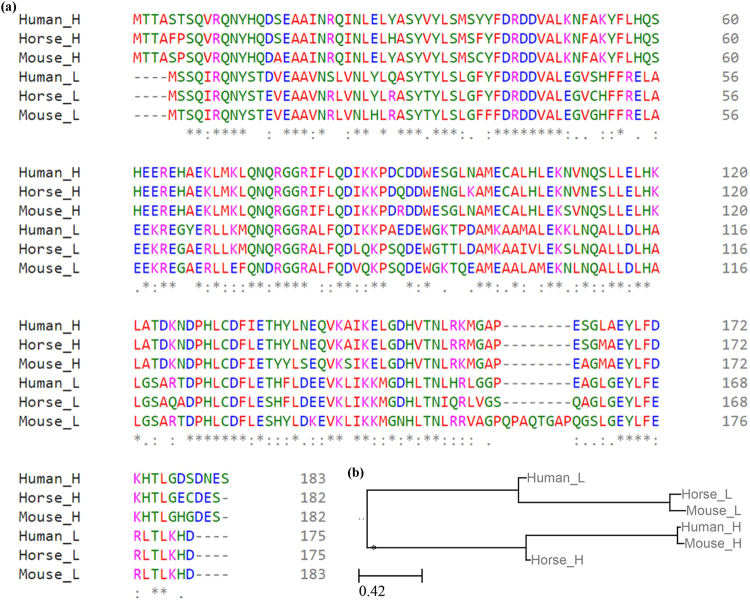
(**a**) Multiple sequence alignment of mouse L-chain (Uniprot database accession P29391), mouse H-chain (Uniprot database accession P09528), human L-chain (Uniprot database accession P02792), human H-chain (Uniprot database accession P02794), horse L-chain (Uniprot database accession P02791) and horse H-chain (Uniprot database accession Q8MIP0) ferritin. (**b**) Phylogenetic tree showing the distance between mouse L-, mouse H-, human L-, human H-, horse L- and horse H-chain ferritins.

Since the APO used in this work was isolated from equine spleen, concerns about its immunogenicity in mice and future human patients can arise. For this reason, sequences of light- and heavy-chain APO from mouse, human and horse were downloaded from Uniprot database and subjected to multiple sequence alignment (Fig. 6a). The L-chain and H-chain APO from these organisms showed very high homology, with most amino acid changes resulting in amino acid with the same charge and only very minor changes to the structure. The phylogenetic tree (Fig. 6b) also shows the high sequence homology.

## Discussion

Out of the 3.4 trillion USD annually spent on health care in the USA only^23^, 30.1 billion dollars is spent towards the impact and management of adverse drug reactions due to the increased need of hospitalization and additional clinical investigations^24^. With its share of 125 billion dollars spent^25^, anti-tumor treatment and its many severe side effects are the cause of a large part of these unneeded extra expenses, not only decreasing the quality of patients’ lives but causing socioeconomic damage as well. The cardiotoxicity of one of the most commonly used chemotherapeutic agents, DOX, was observed in up to 26% of adult patients^4^ and, moreover, in 65% of patients who received treatment in their childhood^26^.

Encapsulation of suboptimal therapeutics inside suitable nano-scaled carriers and their preferential delivery to tumor cells can help overcome these limitations. Nanocarriers not only protect the drug cargo from hostile environments in the body and increase its circulation time but, more importantly, all nanocarriers of 20–200 nm have the ability to selectively accumulate in tumor tissue due to the Enhanced Permeability and Retention (EPR) effect – their abnormal vasculature lacking smooth-muscle layer and containing large fenestrations, no constant blood flow and impaired lymphatic drainage^27^. However, it has lately been discovered that the EPR effect amounts to increased accumulation of nanoparticles in tumor tissue by only 20–30%^28^ and even less in tumors with poor vascularization, such as prostate or metastatic liver cancer^29^. Thus, nanocarriers relying only on this passive way of targeting are still not effective enough. Moreover, even if EPR did significantly improve the accumulation in tumor vicinity, cargo delivery inside the actual cancer cells is not ensured. Still, suitable size is an important parameter in order to avoid premature removal of the drug from patient’s body and increase, even though only slightly, its accumulation in the tumor^30^.

To fully utilize the properties nano-scaled carriers provide, a more effective treatment strategy has lately been exploited, with active targeting to one of the many membrane-bound proteins overexpressed in multiple types of cancer cells, mostly due to their increased metabolic needs^31^. These targets include scavenger receptors type B1^32^, insulin receptors^33^ or folate receptors^34^. Many cancer cells also need increased uptake of iron, as it is important for large number of cellular processes related to energy metabolism, respiration and DNA synthesis. For this purpose, those cancer cells often overexpress receptors for transferrin (iron-transporting protein), and transferrin-conjugated nanocarriers can be effectively used to increase cellular uptake into these cells^10,19,21,35^. The drawback is that metabolically highly active cells also often overexpress these receptors, so the delivery is not as specific as required^36^. Therefore, more specific targets are required. The nanocarrier in the presented study was targeted towards PSMA, a 100 kDa type II membrane glycoprotein, highly expressed on almost all prostate cancer cells^37^ or endothelial cells of tumor vasculature of many non-prostatic solid malignancies but not on healthy cells^38^. Since Liu and coworkers found that anti-PSMA antibodies are constitutively endocytosed in LNCaP cells *via* clathrin-coated pits^39^, we expect endocytosis to be the major internalization route of APODOX-anti-PSMA.

Ferritins are iron storage proteins that can be ubiquitously found in all life forms, except yeast^40^. Once emptied of their iron content, they create a hollow icosahedral protein cage – APO^41^. Their quartenary structure is formed by 24 subunits with various ratios of heavy (H-, 21 kDa) and light (L-, 19 kDa) chain. Based on this H-/L-chain ratio, APO can enter cells *via* TfR (for H-chain APO)^22^, TIM-2 (for H-chain APO)^42,43^ or SCARA5 (for L-chain APO)^22,44,45^, although some works have mistakenly identified TfR as the main receptor for both H- and L-chain APO^46–48^.

The easily performed encapsulation protocol of small molecules in APO is based on its responsiveness to the surrounding pH^49,50^. The quaternary structure of APO reversibly disassembles in pH between 2.0 and 3.4 and reassembles once in a pH above 7.0^51^. The APO-encapsulated drug has a very good stability during long-term storage^52^.

Different approaches to introducing actively targeted moieties onto APO surfaces, have been deployed. Some works relied on natural APO receptors^47,48,53^, others introduced affibodies or short peptides for targeting to tumor cells^6^ or vasculature^54^. We employed targeting *via* site-directed antibodies. APO surface was functionalized by adsorption of gold nanoparticles to multitudes of positively and negatively charged amino acid residues. These adsorbed gold nanoparticles further enabled binding of a heptapeptide derived from protein A (by its cysteine-rich C-terminus). We achieved site-directed orientation of antibodies due to the high affinity of N-terminus of this peptide towards Fc fragment of immunoglobulins (K_D_ = 10 µM for IgG1)^55,56^. This enables a straight facing of paratopes towards antigens, while increasing the immunoefficiency. In non-directed systems, immunoperformance is inhibited by random interactions between paratopes and nanocarrier’s surface causing sterical blockades^17^. In the previously performed *in vitro* toxicological tests, we were able to conclude that PSMA-targeted APO selectively delivered DOX into PSMA-overexpressing cancer cells. This construct inhibited the growth of cancer cells with a similar potency as free DOX while significantly sparing non-malignant cells from the negative effects of DOX^17^.

Numerous nanocarriers tested over the past decades have had very promising *in vitro* results, only to fail, due to multiple reasons, once administered in organism. Some nanocarriers unwantedly interact with biological milieu and their outer surface needs to be modified with polymers or peptides, decreasing these interactions^19,57,58^. Some nanocarriers have high premature efflux of drug molecules in blood, meaning that nanocarriers often reach tumor site as drug-free^30^. Our previous results proved that DOX is not prematurely released from APO during its circulation in bloodstream, but is only removed once in intracellular environment^17^.

To evaluate the *in vivo* effects of PSMA-targeted, APO-encapsulated DOX, xenograft mice were treated with DOX/APODOX/APODOX-anti-PSMA. First, we studied the rate of cellular uptake of these various DOX forms in PSMA-overexpressing cells. Although the highest cellular uptake was observed for free DOX, PSMA-targeting was able to significantly increase the uptake compared to non-modified APODOX. We performed a competitive assay with anti-PSMA antibodies to elucidate that APODOX-anti-PSMA does indeed employ PSMA for the internalization in target cells instead of receptors that are employed by bare ferritins. As horse spleen APO is composed of 22/24 L-chain subunits, it preferentially internalizes into cells through SCARA5^22,44,45,59^. However, since the expression of L-chain ferritin receptor SCARA5 on LNCaP cells was negligible and 8% of horse spleen APO subunits are heavy, we tested the influence of competition with H-chain TfR antibody and proved that this competition had no significant effect on APODOX-anti-PSMA internalization.

Since the protein A-derived heptapeptide used to bind targeting anti-PSMA antibody has affinity towards Fc region of antibodies produced in various organisms^56^, we further confirmed the stability of murine anti-PSMA binding even after prolonged competition with human IgG antibodies.

Next, the attenuation of PSMA-overexpressing tumors in mice treated with these various forms of DOX was evaluated. The fastest attenuation was observed for free DOX, which is in accordance with *in vitro* experiments. However, APODOX and APODOX-anti-PSMA showed similar anti-cancer potency, showing that both of these forms of DOX were able to reach the surface of tumor, likely due to EPR effect. However, after the treatment course ended, the investigation of DOX distribution in tumor slices clearly showed that the DOX was able to reach the inner part of tumor only in mice treated with APODOX-anti-PSMA while in mice treated with APODOX, it only reached the surface of the tumor. This phenomenon can very often be observed for various nanopharmaceuticals where their high concentration on the surface of tumor and its high interstitial pressure can actually reduce tumor perfusion and thus hinder the dose of drug that reaches the entire tumor mass^9^.

Besides easy encapsulation protocol with a high encapsulation efficacy, reliable targeting and convenient size, suitable nanocarrier needs to be completely biocompatible, atoxic and biodegradable^30^. Many nanocarriers that are not natural to patient’s body can cause adverse reactions, accumulate in the body for prolonged time after their cargo is delivered or, upon entering the bloodstream, bind blood proteins, be opsonized and taken up by mononuclear phagocytes, never delivering their cargo at all^60^. Strategies to avoid the immune response often include modification of the nanocarrier surface by stealth molecules, such as PEG. However, these modifications actually hamper the entry to cancer cells and the delivery of the cargo^7^. APO, as a ubiquitously found protein among all life forms with a high sequence homology, is natural for the body. Although ferritin itself is atoxic to the organism, some studies have shown that apoferritin-based nanoformulations can negatively influence iron metabolism, i. e. remove iron from off-target tissues^61^, due to its higher natural affinity for iron than for the encapsulated drug molecules^15^. In this work, we proved that the iron levels in off-target organs remained intact in APODOX-anti-PSMA-treated mice, although some changes were observed in mice treated with either DOX or APODOX. This proved that DOX-loaded and site-directed APODOX does not negatively influence iron metabolism.

To investigate the non-specific toxicity of various types of DOX, the effect of the treatment on off-target tissues was also studied, namely heart, liver and kidney, which are most prominently damaged by DOX. While DOX treatment showed significant influence on the body weight of treated mice, no such trend was observed for either of the APO-encapsulated DOX formulations.

On the organ level, biochemical analyses and histological assessment clearly showed damage to liver of mice treated with DOX and non-targeted APODOX, as evidenced by the elevation in levels of liver enzymes, congested central veins and hepatocyte binucleations. Although small number of hepatocyte binucleations (as well as DOX presence in boundaries of the lobules) was also observed in mice treated with APODOX-anti-PSMA, the levels of liver enzymes showed no damage to the liver as a whole.

Furthermore, no significant increase in the levels of kidney enzymes and no DOX distribution in kidney were observed in any of the treated groups, showing functional glomerular filtration. However, large number of intraluminal protein casts was found in DOX-treated mice. APODOX- and APODOX-anti-PSMA-treated mice also showed the formation of these protein casts, although their number was much lower, proving lower damage to kidney compared to that caused by free DOX.

In heart, the organ which is clinically damaged by DOX treatment the most, higher DOX concentration was found in the myocardium of mice treated with DOX and APODOX, as well as vacuolation of the myocardiocytes. The observed toxicity of non-targeted APODOX could be explained by its affinity to SCARA5, which is also expressed by heart muscle cells^62^. APODOX-anti-PSMA-treated mice had negligible amounts of myocardiocyte vacuolation and very low DOX concentration was observed in their myocardium.

## Material and Methods

### Chemicals

All chemicals were obtained in ACS purity from Sigma-Aldrich (St. Louis, MO, USA), unless otherwise stated. The pH was measured using pH meter WTW inoLab (Weilheim, Germany). Solution of 0.2 µm-filtered horse spleen APO in 0.135 M sodium chloride (cat. no. A3641), as well as doxorubicin hydrochloride (cat. no. 44583) were purchased from Sigma-Aldrich.

### Synthesis of components and assembly of APODOX and APODOX-anti-PSMA

APODOX was prepared following the protocol published in our previous study^17^. Briefly, 200 µL of 1 mg∙mL^−1^ DOX (in water) was added to 20 µL of 50 mg∙mL^−1^ horse spleen APO and 100 µL of water. Hydrochloric acid (2.5 µL, 1 M) was added to decrease the pH of the solution to 2.7 and disassociate the APO. The solution was stirred for 15 min to create a homogeneous mixture of APO and DOX molecules. Sodium hydroxide (2.5 µL, 1 M) was added to increase the pH to 6.5 and physically entrap the DOX molecules in APO cavity (creating APODOX). The mixture was kept at 20 °C for 15 min. To remove the non-encapsulated DOX molecules, water exchange was performed 3× by diafiltration of loaded APODOX through Amicon 3 K filters (Merck Millipore, Burlington, MA, USA), with centrifugation at 6000 *g* and 4 °C for 15 min each time.

To achieve the prostate cancer-targeting attributes, the surface of APODOX was first modified with gold nanoparticles (AuNPs) prepared as described in our previous work^17^. Briefly, 2 mL of 1% trisodium citrate was added to 10 mL of 1 mM gold(III) chloride hydrate and shaken on Orbital Shaker (Biosan, Riga, Latvia) at 20 °C for 72 h. The resulting AuNPs were 1.3 nm in diameter. To APODOX, 25 µL of 1 mM solution of AuNPs was added and the mixture was shaken at 20 °C for 12 h (creating APODOX-Nano). The unbound AuNPs were removed by 2× water exchange as decribed above. Next, 2.8 µL of 1250 µg∙mL^−1^ Protein A-derived HWR peptide-based linker (HWRGWVC) was synthesized on the solid phase with CEM Liberty Blue Synthesizer (Matthews, NC, USA) from 9H-fluoren-9-yl methoxy carbonyl**-**protected precursors. Its purity was evaluated using HPLC-UV (ESA Inc., Chelmsford, MA, USA) and the molecular weight was verified by HPLC-ESI-QqTOF (Bruker Daltonik GmBH, Bremen, Germany). HWR peptide was added to APODOX-Nano and the mixture was incubated at 45 °C and 400 rpm for 1 h (to achieve the binding of the HWRGWVC linker; see our previous study^17^). Unbound HWR peptide was removed by water exchange as described above. Mouse monoclonal anti-PSMA antibodies GCP-05 (ab66912, Abcam, Cambridge, UK; 17.5 ng) were added to the samples to achieve complete binding to HWR peptide and the mixture was incubated at 20 °C and 600 rpm for 1 h, creating APODOX-anti-PSMA^17^. DOX concentration in the nanocarrier was evaluated using its specific absorbance at 480 nm measured on Tecan Infinite 200 PRO (Tecan, Männedorf, Switzerland). The size and ζ-potential measurements of the nanocarriers, as well as their visualization were performed according to^17,52^.

To show the protein content, the samples were resolved at 200 V for 35 min on 12.5% Tris/Glycine native or SDS-PAGE (polyacrylamide gel electrophoresis) using a continuous buffer system. The samples were loaded with a loading buffer in 2:1 ratio and the gels were stained with coomassie blue. As a molecular weight marker, Precision Plus Protein^TM^ Dual Xtra Prestained Protein Standard (cat. no. 1610377, Bio-Rad, Hercules, CA, USA) was used.

### Cell lines and cell culturing conditions

Human cell lines LNCaP (CRL-1740^TM^, derived from left supraclavicular androgen-dependent lymph node prostate cancer metastasis) and 22RV1 (CRL-2505^TM^, derived from a xenograft serially propagated in mice after castration-induced regression and relapse of the parental androgen-dependent CWR22 xenograft) were purchased from the American Type Culture Collection (Manassas, VA, USA). The cells were maintained with RPMI Medium 1640 (1×) + GlutaMAX^TM^ (Gibco, Waltham, MA, USA) supplemented with 10% fetal bovine serum (Gibco, Waltham, MA, USA) and 1× antibiotics mix ZellShield (Minerva Biolabs GmbH, Berlin, Germany). Cells were incubated in Galaxy 170 R (Eppendorf AG, Hamburg, Germany) with 5% CO_2_ in the air at 37 °C.

### Protein extraction, western blotting and antibody-conjugated nanoparticles assay (Affi-assay)

Quantitative expression of PSMA, TfR and SCARA5 on LNCaP and 22RV1 cells was studied using western blot. The cells were lysed with RIPA buffer and the lysate proteins were separated on 12.5% SDS-PAGE gel. The proteins were transferred to the Immun-Blot^®^ PVDF membrane (Bio-Rad, Hercules, CA, USA). Anti-GAPDH antibody G-9 (sc-365062, Santa Cruz Biotechnology, Dallas, TX, USA), Anti-Transferrin Receptor 1 antibody 13E4 (ab38171, Abcam, Cambridge, UK), Anti-SCARA5 antibody (ab118894, Abcam, Cambridge, UK) and anti-PSMA antibody (ab66912, Abcam, Cambridge, UK) were used, diluted in antibody buffer [1 mg∙mL^−1^ BSA in phosphate buffered saline (PBS)] in 1:700, 1:1000, 1:1000 and 1:500 ratio, respectively. To evaluate the binding of APODOX-anti-PSMA to target antigen, the Affi-assay was performed as follows: after transfer of cell proteins to PVDF membrane and blocking with 1% milk, the membrane was rinsed with PBS and incubated overnight in APODOX-anti-PSMA (DOX concentration of 316 µM). DOX fluorescence was detected using excitation at 550 nm and emission at 570 nm using Azure c600 (Azure Biosystems, Dublin, CA, USA). The individual spots were cropped from different parts of the same membrane and divided with white space using Microsoft Office Powerpoint software (Redmond, WA, USA). All densitometric analyses were performed on uncropped membranes using AzureSpot software (Azure Biosystems, Dublin, CA, USA).

### Analysis and quantitation of internalization into prostate cancer cells

To determine the rate of cellular uptake of DOX/APODOX/APODOX-anti-PSMA, the quantitative phase imaging was performed by Tescan multimodal holographic microscope Q-PHASE, based on the original concept of coherence-controlled holographic microscope. Cells (1 × 10^4^) were cultivated in Flow chambers μ-Slide I Lauer Family (Ibidi, Martinsried, Germany). Holograms were captured by CCD camera (MR4021 MC-Veleta, Ximea, Münster, Germany). Quantitative phase images are shown in grayscale with units of pg∙μm^−2^ that were recalculated from original radians according to Barer and Davies^63,64^. To determine the mechanism of intracellular uptake, 1 × 10^5^ cells in 1 mL of medium were seeded in each of 4 wells in 12-well culture plate and cultivated for 21 h. After cultivation, the medium was discarded and replaced with 100 µL of fresh medium containing 34 µM DOX/APODOX/APODOX-anti-PSMA, followed by incubation for 24 h. To evaluate the competitive inhibitory effects of anti-PSMA and anti-TfR, the cells were pretreated with 10 µg∙mL^−1^ of these antibodies at 37 °C for 30 min prior to the treatment. After treatment, the cells were washed with 200 μL of PBS and microscoped.

### Plasma stability of APODOX-anti-PSMA

To test the stability of anti-PSMA antibody binding to APODOX surface, APODOX-anti-PSMA was mixed with IgG antibodies from human serum (cat. no. I4506–10MG) at plasma concentration (10 mg∙mL^−1^) and incubated at 37 °C and 600 rpm. At various time points (0, 0.5, 24 and 48 h), the mixture was centrifuged at 6000 *g* and 4 °C for 15 min, and the pellet containing the nanocarrier was resuspended in Ringer’s solution (0.65% NaCl, 0.042% KCl, 0.025% CaCl_2_, 0.02% sodium bicarbonate). The samples were separated on 12.5% SDS-PAGE gel and transferred to the Immun-Blot^®^ PVDF membrane. To evaluate the binding of mouse anti-PSMA antibodies, rabbit anti-mouse/HRP antibody (P0260, Thermo Fisher Scientific Inc., Waltham, MA, USA) diluted in antibody buffer in 1:5000 ratio was used, followed by chemiluminescent detection using Clarity^TM^ Wester ECL Substrate (Bio-Rad).

### Induction of prostate tumor xenografts and treatment protocol

Twelve five-week-old male nude athymic BALB/c nu/nu mice were used for xenograft studies. The use of the animals followed the European Community Guidelines as accepted principles for the use of experimental animals. The experiments were performed with the approval of the Ethics Commission at the Faculty of Medicine, Masaryk University, Brno, Czech Republic. The mice were housed in individually ventilated cages at 12/12 h light/dark cycle and provided *ad libitum* with standard diet and water. LNCaP cells (5 × 10^6^) were resuspended in 100 µL of PBS with 20% Matrigel (*v/v*, BD Biosciences, Franklin Lakes, NJ, USA) and implanted subcutaneously into the right flank region of the mice under general anesthesia (1% Narkamon +2% Rometar, 5 µL∙g^−1^ of body weight). The treatment of mice was carried out intravenously (through tail vein) once a week for 21 days (total of 4 applications) using 5 µg∙g^−1^ of body weight of DOX, either free or in the form of APODOX or APODOX-anti-PSMA. The control group received 100 µL of 0.9% sodium chloride (saline). Changes in tumor volume were recorded bidaily using the contactless measuring device Peira TM900 (Peira, Turnhout, Belgium).

After 3 weeks of treatment, the mice were euthanized by intraperitoneal injection of 1% Narkamon +2% Rometar, 5 µL∙g^−1^ of body weight, followed by intracardiac blood collection in ethylendiaminetetraacetic acid (EDTA)-treated tubes. Plasma was collected and subjected to biochemical analyses using automated spectrophotometer BS-400 (Mindray, Schenzhen, China). The tumor, heart, kidneys, and liver were collected, snap-freezed and stored at −80 °C prior to further experiments.

### Histological procedures and assessment of excised organs

The tumor, heart, kidneys, and liver of each mouse were investigated to assess tissue microstructure, DOX distribution and iron distribution. The samples were fixed in formaldehyde (10%, *v/v*) overnight, subsequently dehydrated in a series of progressively more concentrated ethanol and embedded in paraffin wax. Sections were cut at 5 µm, mounted on glass slides, deparaffinized and either subjected to fluorescent detection of DOX distribution or stained with hematoxylin-eosin for assessment of structure and Perls’ Prussian blue for detection of iron. The microscopical observations were conducted using Olympus IX 71S8F-3 (Olympus, Tokyo, Japan).

Quantitative DOX detection was performed *via* the acidified isopropanol extraction according to a previously reported procedure^20^. Briefly, approx. 0.02 g of tumor, heart, liver and kidney collected from all groups was homogenized with an addition of 1:10 (wt/vol) acidified (0.75 M HCl) isopropanol on ice and extracted overnight at −20 °C in the dark. The samples were then centrifuged at 4 °C and 18000 *g* for 10 min. DOX fluorescence (excitation at 480 nm, emission at 600 nm) was measured in the supernatant with correction of tissue autofluorescence using the homogenates from saline-injected controls. The results were presented as µg per gram of tissue and expressed as means ± SD for a group ($n=3$ mice per group).

### Descriptive statistics and data processing

Results are expressed as mean ± standard deviation unless noted otherwise. Statistical analysis was performed with two-tailed Student’s *t*-test between two groups. One-way ANOVA was conducted to assess significance among multiple groups, followed by two-tailed Student’s *t*-test if *p* < 0.05. Software Statistica 12 (StatSoft, Tulsa, OK, USA) was employed for analyses.

Mouse L- (P29391), mouse H- (P09528), human L- (P02792), human H- (P02794), horse L- (P02791), and horse H- (Q8MIP0) ferritin sequences were downloaded from the Uniprot database and the sequence alignment was performed using CLUSTAL Omega (v. 1.2.4.) multiple sequence alignment. The phylogenetic tree was constructed using Blast Tree View tool according to Neighbor joining method and default settings (max Seq difference 0.85 and Distance according to Grishin for proteins). The central midpoint root is shown on tree as (*). http://etetoolkit.org/treeview/ using the Newick format (tree.nwk in attachment) was used to make simpler tree graphics.

All schematics and figures were processed using Microsoft Office PowerPoint software (Redmond, WA, USA), unless otherwise mentioned. The schematics of mouse and syringe were adopted under General Public Licence from pixabay.com.

### Data availability

The datasets generated during and/or analysed during the current study are available from the corresponding author on reasonable request.

## Electronic supplementary material

Video 1

Video 2

Video 3

Video 4

Video 5

Captions for Supplementary Videos
