# Supplementary material for: Prostate-Specific Membrane Antigen-Targeted Site-Directed Antibody-Conjugated Apoferritin Nanovehicle Favorably Influences In Vivo Side Effects of Doxorubicin
Source: Sci Rep. 2018 Jun 11;8:8867. doi: 10.1038/s41598-018-26772-z (PMC5995913; doi:10.1038/s41598-018-26772-z)
Supplement: Supplementary file 6 — Captions for Supplementary Videos [file 41598_2018_26772_MOESM6_ESM.pdf]

Prostate-Specific Membrane Antigen-Targeted Site-Directed Antibody-Conjugated Apoferritin Nanovehicle Favorably Influences *In Vivo* Side Effects of Doxorubicin

Simona Dostalova[†,⌈], *Hana Polanska*[Ŧ], *Marketa Svobodova*[Ŧ], *Jan Balvan*[Ŧ,↑], *Olga Krystofova*[†,⌈], *Yazan Haddad*[†,⌈], *Sona Krizkova*[†,⌈], *Michal Masarik*[Ŧ], *Tomas Eckschlager*[⁝], *Marie Stiborova*[&], *Zbynek Heger*[†,⌈], *Vojtech Adam*[†,⌈]*

[†]Department of Chemistry and Biochemistry, Mendel University in Brno, Zemedelska 1, Brno CZ-613 00, Czech Republic

[⌈]Central European Institute of Technology, Brno University of Technology, Purkynova 123, Brno CZ-612 00, Czech Republic

[Ŧ]Department of Pathological Physiology, Faculty of Medicine, Masaryk University, Kamenice 753/5, Brno CZ-625 00, Czech Republic

[↑]TESCAN ORSAY HOLDING a.s., Libusina trida 863/21, Brno CZ-623 00, Czech Republic

[⁝]Department of Pediatric Hematology and Oncology, 2nd Faculty of Medicine, Charles University and University Hospital Motol, V Uvalu 84/1, Prague 5 CZ-150 06, Czech Republic

[&]Department of Biochemistry, Faculty of Science, Charles University, Hlavova 2030/8, Prague 2 CZ-128 43, Czech Republic

Supporting Information Titles

Video 1 - Time lapse video of DOX uptake

Video 2 - Time lapse video of APODOX uptake

Video 3 - Time lapse video of APODOX-anti-PSMA uptake

Video 4 - Time lapse video of uptake of APODOX-anti-PSMA in competition setup using anti-TfR antibodies

Video 5 - Time lapse video of uptake of APODOX-anti-PSMA in competition setup using anti-PSMA antibodies
